# Regional patterns and trends of hearing loss in England: evidence from the English longitudinal study of ageing (ELSA) and implications for health policy

**DOI:** 10.1186/s12877-020-01945-6

**Published:** 2020-12-15

**Authors:** Dialechti Tsimpida, Evangelos Kontopantelis, Darren M. Ashcroft, Maria Panagioti

**Affiliations:** 1 Centre for Primary Care and Health Services Research, Institute for Health Policy and Organisation (IHPO), School of Health Sciences, Faculty of Biology, Medicine and Health, 5th floor Williamson Building, The University of Manchester, Oxford Road, Manchester, M139PL UK; 2 Institute for Health Policy and Organisation (IHPO), School of Health Sciences, Faculty of Biology, Medicine and Health, The University of Manchester, Manchester, UK; 3 NIHR Greater Manchester Patient Safety Translational Research Centre, School of Health Sciences, Faculty of Biology, Medicine and Health, The University of Manchester, Manchester, UK

**Keywords:** Hearing loss, ELSA, inequalities, Social epidemiology, Health geography

## Abstract

**Background:**

Hearing loss (HL) is a significant public health concern globally and is estimated to affect over nine million people in England. The aim of this research was to explore the regional patterns and trends of HL in a representative longitudinal prospective cohort study of the English population aged 50 and over.

**Methods:**

We used the full dataset (74,699 person-years) of self-reported hearing data from all eight Waves of the English Longitudinal Study of Ageing (ELSA) (2002–2017). We examined the geographical identifiers of the participants at the Government Office Region (GOR) level and the geographically based Index of Multiple Deprivation (IMD). The primary outcome measure was self-reported HL; it consisted of a merged category of people who rated their hearing as fair or poor on a five-point Likert scale (excellent, very good, good, fair or poor) or responded positively when asked whether they find it difficult to follow a conversation if there is background noise (e.g. noise from a TV, a radio or children playing).

**Results:**

A marked elevation in HL prevalence (10.2%) independent of the age of the participants was observed in England in 2002–2017. The mean HL prevalence increased from 38.50 (95%CI 37.37–39.14) in Wave 1 to 48.66 (95%CI 47.11–49.54) in Wave 8. We identified three critical patterns of findings concerning regional trends: the highest HL prevalence among samples with equal means of age was observed in GORs with the highest prevalence of participants in the most deprived (IMD) quintile, in routine or manual occupations and misusing alcohol. The adjusted HL predictions at the means (APMs) showed marked regional variability and hearing health inequalities between Northern and Southern England that were previously unknown.

**Conclusions:**

A sociospatial approach is crucial for planning sustainable models of hearing care based on actual needs and reducing hearing health inequalities. The Clinical Commissioning Groups (CCGs) currently responsible for the NHS audiology services in England should not consider HL an inevitable accompaniment of older age; instead, they should incorporate socio-economic factors and modifiable lifestyle behaviours for HL within their spatial patterning in England.

**Supplementary Information:**

The online version contains supplementary material available at 10.1186/s12877-020-01945-6.

## Background

Hearing loss (HL) is a significant public health concern that costs the UK economy £25 billion a year in productivity and unemployment [1], an amount that equates to one-fifth of the total annual health spending in England in 2018/19 [2]. HL affects over nine million people in England, and it is estimated that, by 2035, the number of people with HL will rise to around 13 million. The above estimates, along with the local hearing needs in England, are calculated by population projections based on the study of Davis [3], who collected and analysed audiological data in the 1980s. This study remains the primary source of local estimates of HL prevalence [4]; recently, these estimates have also been visualised in the form of a hearing map, offering a rough guide to the prevalence of HL among adults across the UK [5].

Despite its importance to the history of hearing care in the UK, Davis’s study had some significant limitations. First, the English samples were solely derived from the cities of Nottingham and Southampton, which are very unlikely to be representative of the whole population of England [3]. The role of place in health is well-established [6, 7], and research has shown that it affects health outcomes [6]. Second, scientific thinking in HL research was formed in previous decades around the concepts of older age and the male sex being the main leading causes of HL in adults, with little or no consideration for modifiable risk factors for hearing acuity. However, recent findings have suggested that socio-economic factors and modifiable lifestyle behaviours are associated with the likelihood of HL as firmly as well-established demographic factors such as age and sex [8]. Thus, the study of Davis did not consider in its estimations the effects of place and socio-economic factors such as high occupational noise exposure from manual occupations [9] and differences in regions with strong and weak manufacturing industries [10].

The Clinical Commissioning Groups (CCGs) are currently responsible for the NHS audiology services in England, including the provision of hearing aids [11]. However, the lack of robust hearing data makes it difficult to plan efficient, effective and sustainable models of hearing care based on patient needs [10]. Exploratory spatial data analysis of hearing data from a representative population sample in England would reveal regional patterns and trends of HL, shedding light on potential socio-economic inequalities in hearing health. This updated analysis of HL prevalence could inform the health policy strategies of the NHS England and Department of Health, particularly in respect of the new governmental programme, ‘Action Plan on Hearing Loss’ [1].

The aim of this study was, therefore, to explore regional patterns and trends of HL in a representative longitudinal prospective cohort study of the English population aged 50 and over.

## Methods

### Study population

The study utilised data from the English Longitudinal Study of Ageing (ELSA). The ELSA is a longitudinal prospective cohort study that collects multidisciplinary data from a nationally representative sample of community-dwelling middle-aged and older (aged 50 and above) adults in England [12]. The study started in 2002 and is collecting responses every 2 years on participants’ health, social, wellbeing and economic circumstances. The current sample contains data from eight Waves, covering the period 2002–2017 [13]. As the ELSA follows a longitudinal design, the sample is comprised of a sequence of observations on the same individuals across Waves and the refreshment samples (Cohorts 3, 4, 6 and 7) [13]. Proxy interviews were carried out in case an ELSA panel member refused to further participate [14]. In our analyses, we used the full dataset (74,699 person-years) of self-reported hearing data from all eight Waves of the ELSA.

The ELSA follows the sampling strategy of the Health Survey for England (HSE), which ensures that every address on the small users’ Postcode Address File (PAF) in England has an equal chance of inclusion. Field household contact rates of over 96% were achieved. The study excluded cases not belonging to the target population through ‘terminating events’, such as deaths, institutional moves and moves out of England since taking part in the HSE [15].

### Outcomes

#### Hearing acuity

Self-rated hearing data was collected from participants across all Waves. According to the study’s documentation, self-reported HL was defined as declarations of fair or poor hearing on a five-point Likert scale (excellent, very good, good, fair or poor) or ‘Yes’ responses to the question concerning whether or not the participants find it difficult to follow a conversation if there is background noise (e.g. noise from a TV, a radio or children playing) [13, 16].

#### Geographical variables

The geographically related information of the ELSA dataset was in the form of identifiers such as the Government Office Region (GOR) [17], and indices that are used as measure of poverty of different geographical areas, such as the Index of Multiple Deprivation (IMD). The geographical variables were provided to the first author under a Special License and Secure Access agreement (UK Data Service Project Number: 121175).

Each respondent’s geography is determined by their residence postcode at the time of the survey interview date. Different versions of the IMD were provided for the eight Waves of the ELSA: IMD 2004 [18] for Waves 1–3, IMD 2007 [19] for Wave 4, IMD 2010 [20] for Waves 5–7 and IMD 2015 [21] for Wave 8. The IMD was provided in quintiles (the first quintile being the least deprived, the fifth being the most deprived).

The nine GORs represent the highest tier of sub-national division in England (North East, North West, Yorkshire and the Humber, East Midlands, West Midlands, East of England, London, South East, South West).

#### Covariates

For covariates, we examined non-modifiable factors (age, sex), partly modifiable indicators of socio-economic position (SEP) (education, occupation, income, wealth) and alcohol consumption as a fully modifiable lifestyle risk factor for HL. Age was assessed both as a discrete (as only certain values could be taken) and categorical variable in three groups (50–64, 65–74, 75–89). We used this categorisation to allow for a comparison with Benova et al. [22], who examined the association of SEP with self-reported hearing difficulties in Wave 2 of the ELSA.

We considered five categories regarding highest educational attainment: no qualifications, foreign or other, O level Certificate of Secondary Education, A level (Level 3 Qualification of the National Qualifications Framework) and a degree or higher education.

Tertiles of self-reported occupation were based on the National Statistics Socio-economic Classification (NS-SEC): routine and manual occupations; intermediate; managerial and professional. The relative financial position of the participants was captured by quintiles of net household income (the first quintile being the lowest, the fifth being the highest). Wealth was examined in quintiles of the net total non-pension wealth reported at the household unit level (the first quintile being the highest, the fifth being the lowest).

Alcohol consumption was selected as the only lifestyle factor that was consistently recorded in all Waves. We constructed a continuous variable to represent the sum of units of alcohol that each participant consumed during the last 7 days. This variable was dichotomised into those that consumed more than 14 units of alcohol in the last 7 days and those that did not, using the Chief Medical Officer’s Drinking Guidelines [23].

#### Data analysis

Categorical variables are presented as absolute (n) and relative (%) frequencies, while continuous variables are presented through their mean and standard deviation. We used the full dataset from the eight Waves (74,699 person-years) to strengthen the argument that there is a correlation between spatial variables and HL over time. A small number of cases (one in Wave 0 and eight in Wave 2) in the geographical identifiers had missing values because the address was located within Wales (which uses its own deprivation index). Due to the low proportion of missingness in the variables, records with missing data were excluded from analyses (3.2% of all records in listwise deletion). We used Bartlett’s test for homogeneity of variances to test that age variances were equal for all samples. Following this, we applied one-way analysis of variance (ANOVA) to compare the means of age among GOR samples in all Waves. We also computed adjusted predictions at the means (APMs) and the marginal effects at the means (MEMs) [24] for the HL prevalence in each Wave of the ELSA, with age, sex, education, occupation, income, wealth, IMD and alcohol consumption as the factor variables.

We used local spatial analysis statistical tools for analysing spatial distributions, patterns, processes and relationships in the geographical data. We used the Spatial Join tool to aggregate the number of cases of self-reported HL to total responses of hearing acuity in each polygon (GOR) in order to visualise the prevalence rates of HL per GOR. We used the Natural Breaks (Jenks) classification to optimise the arrangement of the sets of HL values into ‘natural’ classes, a method also known as the goodness of variance fit (GVF). Furthermore, we used the Hot Spot Analysis (Getis-Ord Gi*) as a mapping cluster tool to identify the locations of statistically significant Hot Spots and Cold Spots. The Getis-Ord Gi* is an inferential statistic for the conceptualisation of spatial relationships, used when one is looking for unexpected spatial spikes of high values. In essence, this tool works by looking at each feature within the context of neighbouring features and assessing whether high or low values cluster spatially. Due to the small scale of the analysis, we chose this local spatial statistic tool so that the value of each feature could be included in its own analysis, along with the neighbouring features.

The Getis-Ord local statistic is given as:

1 $$ {G}_i^{\ast }=\frac{\sum_{j=1}^n{w}_{i,j}{x}_j-\overline{X}{\sum}_{j=1}^n{w}_{i,j}}{\sqrt[S]{\frac{\left[n{\sum}_{j=1}^n{w}_{i,j}^2-{\left({\sum}_{j=1}^n{w}_{i,j}\right)}^2\right]}{n-1}}} $$

Here, *x*_*j*_ is the attribute value for feature *j*, *w*_*i*, *j*_ is the spatial weight between feature *i* and *j*, *n* is equal to the total number of features and:

2 $$ \overline{X}=\frac{\sum_{j=1}^n{x}_j}{n} $$

3 $$ S=\sqrt{\frac{\sum_{j=1}^n{x}_j^2}{n}-{\left(\overline{X}\right)}^2} $$

The ${G}_i^{\ast }$ statistic is a *z*-score, so no further calculations are required.

The spatial relationship was defined according to the ‘Contiguity Edges Corners’, a method that was selected in order to allow all neighbouring polygon features that share a boundary or node to influence the target polygon feature’s computations. *Confidence* levels of *90*, 95 and 99% were considered in the calculations of Getis-Ord Gi*. Data were analysed using Stata version 14 [25] and ESRI ArcGIS Desktop 10.7.1 [26].

## Results

The results of one-way ANOVA indicated that the null hypothesis was not rejected in Waves 2, 6, 7 and 8 (as *p* > 0.05), which means that there is sufficient evidence to conclude that the means of age among GORs’ samples were equal [27]. In addition, the means of age across Waves were significantly equal for all samples (*p* = 0.996). Using Bartlett’s test, we found that the variances of the means of age among GORs were equal in Waves 3, 5, 6, 7 and 8 and across all Waves. A table presenting the one-way ANOVA test results – including sums of squares, mean squares, degrees of freedom and the F-values and *p*-values of means of age across the nine GORs in eight Waves of the ELSA – is provided in Additional File 1.

Table 1 shows the participants’ non-modifiable demographic factors and HL prevalence in England in eight Waves of the ELSA. We observed considerable variation in the prevalence rate of HL among GORs (normalised per GOR population), which reached 12.3%. In Wave 5, the prevalence of HL was 39.55 in the South East (95%CI 37.12–42.04) versus 51.85 in the North East (95%CI 47.66–56.02).

**Table 1:** Participants’ non-modifiable demographic factors and hearing loss prevalence in England in 8 Waves of English Longitudinal Study of Ageing (ELSA)

GOR^**a**^	Hearing loss^**b**^	Men	Women	Age 50–64	Age 65–74	Age 75–89	Mean age (SD)^**c**^	Hearing loss^**b**^	Men	Women	Age 50–64	Age 65–74	Age 75–89	Mean age (SD)
	**WAVE 1 (2002–2003)**							**WAVE 2 (2004–2005)**						
North East	325 (42.54)	323 (41.79)	450 (58.21)	366 (49.80)	245 (33.33)	124 (16.87)	64.33 (10.53)	254 (43.34)	251 (42.33)	342 (57.67)	272 (47.06)	189 (32.70)	117 (20.24)	65.79 (9.97)
North West	603 (38.78)	692 (43.88)	885 (56.12)	799 (53.30)	410 (27.35)	290 (19.35)	64.24 (10.95)	432 (36.86)	517 (43.34)	676 (56.66)	551 (48.25)	337 (29.51)	254 (22.24)	66.21 (10.91)
Yorkshire and The Humber	549 (43.06)	573 (44.28)	721 (55.72)	646 (52.73)	351 (28.65)	228 (18.61)	63.84 (10.91)	401 (40.46)	438 (43.63)	566 (56.37)	474 (48.77)	294 (30.25)	204 (20.99)	65.57 (10.39)
East Midlands	449 (39.18)	528 (45.21)	640 (54.79)	605 (55.50)	297 (27.25)	188 (17.25)	63.30 (11.06)	335 (35.52)	425 (44.41)	532 (55.59)	481 (2.17)	247 (26.79)	194 (21.04)	64.99 (10.42)
West Midlands	452 (36.78)	550 (43.69)	709 (56.31)	599 (49.92)	358 (29.83)	243 (20.25)	64.77 (10.99)	375 (38.82)	422 (43.28)	553 (56.72)	445 (47.34)	290 (30.85)	205 (21.81)	66.06 (10.64)
East of England	507 (37.50)	596 (43.95)	760 (56.05)	663 (51.88)	368 (28.79	247 (19.33)	64.19 (11.16)	400 (37.00)	491 (44.84)	604 (55.16)	507 (48.33)	317 (30.22)	225 (21.45)	65.81 (10.85)
London	385 (34.16)	484 (42.31)	660 (57.69)	574 (53.75)	268 (25.09)	226 (21.16)	64.46 (11.54)	292 (35.39)	347 (41.61)	487 (58.39)	406 (50.94)	222 (2.85)	169 (21.20)	66.05 (11.22)
South East	681 (36.13)	823 (43.04)	1089 (56.96)	951 (52.57)	484 (26.76)	374 (20.67)	64.27 (10.97)	513 (35.19)	628 (42.58)	847 (57.42)	688 (48.38)	391 (27.50)	343 (24.12)	66.14 (10.79)
South West	510 (38.37)	602 (44.83)	741 (55.17)	605 (47.79)	363 (28.67)	298 (23.54)	65.12 (11.38)	420 (39.77)	468 (43.90)	598 (56.10)	462 (44.90)	307 (29.83)	260 (25.27)	66.64 (10.61)
	**WAVE 3 (2006–2007)**							**WAVE 4 (2008–2009)**						
North East	284 (47.81)	259 (42.81)	346 (57.19)	289 (49.49)	160 (27.40)	135 (23.12)	65.51 (10.67)	284 (47.81)	274 (41.96)	379 (58.04)	306 (48.19)	185 (29.13)	144 (22.68)	66.53 (10.50)
North West	458 (40.39)	508 (43.76)	653 (56.24)	593 (54.45)	276 (25.34)	220 (20.20)	64.53 (11.41)	458 (40.39)	586 (44.46)	732 (55.54)	682 (53.41)	352 (27.56)	243 (19.03)	65.15 (10.57)
Yorkshire and The Humber	452 (42.68)	475 (43.70)	612 (56.30)	546 (53.37)	275 (26.88)	202 (19.75)	64.16 (11.16)	452 (42.68)	492 (42.89)	655 (57.11)	573 (52.04)	322 (29.25)	206 (18.71)	64.95 (10.41)
East Midlands	368 (37.98)	440 (44.44)	550 (55.56)	537 (57.19)	227 (24.17)	175 (18.64)	63.43 (10.95)	368 (37.98)	496 (44.36)	622 (55.64)	597 (55.28)	294 (27.22)	189 (17.50)	64.88 (10.34)
West Midlands	392 (39.68)	435 (43.28)	570 (56.72)	503 (52.89)	243 (25.55)	205 (21.56)	65.13 (11.64)	392 (39.68)	511 (44.40)	640 (55.60)	565 (50.81)	314 (28.24)	233 (20.95)	66.00 (10.71)
East of England	461 (40.33)	524 (44.86)	644 (55.14)	585 (52.89)	297 (26.85)	224 (20.25)	64.42 (11.48)	461 (40.33)	596 (45.05)	727 (54.95)	667 (52.73)	391 (30.91)	207 (16.36)	64.96 (10.44)
London	348 (40.65)	356 (40.78)	517 (59.22)	451 (55.41)	199 (24.45)	164 (20.15)	64.77 (12.06)	348 (40.65)	404 (43.44)	526 (56.56)	487 (54.90)	242 (27.28)	158 (17.81)	65.44 (11.09)
South East	534 (35.77)	654 (42.63)	880 (57.37)	767 (52.86)	371 (25.57)	313 (21.57)	64.84 (11.42)	534 (35.77)	781 (43.51)	1014 (56.49)	902 (52.17)	496 (28.69)	331 (19.14)	65.28 (10.35)
South West	453 (43.06)	476 (44.61)	591 (55.39)	495 (48.82)	266 (26.23)	253 (24.95)	65.81 (11.51)	453 (43.06)	530 (44.06)	673 (55.94)	561 (48.32)	351 (30.23)	249 (21.45)	66.14 (10.54)
	**WAVE 5 (2010–2011)**							**WAVE 6 (2012–2013)**						
North East	295 (51.85)	255 (43.29)	334 (56.71)	241 (41.70)	177 (30.62)	160 (27.68)	66.78 (12.69)	249 (50.00)	226 (43.38)	295 (56.62)	185 (36.42)	167 (32.87)	156 (30.71)	66.97 (14.74)
North West	481 (42.68)	531 (44.81)	654 (55.19)	575 (49.74)	338 (29.24)	243 (21.02)	65.03 (12.99)	471 (46.96)	481 (45.00)	588 (55.00)	442 (42.14)	359 (34.22)	248 (23.64)	66.61 (12.61)
Yorkshire and The Humber	457 (45.11)	442 (42.34)	602 (57.66)	484 (47.78)	315 (31.10)	214 (21.13)	65.29 (12.26)	453 (48.81)	407 (42.05)	561 (57.95)	401 (42.57)	305 (32.38)	236 (25.05)	66.58 (12.54)
East Midlands	399 (39.66)	467 (44.18)	590 (55.82)	522 (50.68)	297 (28.83)	211 (20.49)	64.85 (12.34)	391 (41.68)	430 (43.61)	556 (56.39)	406 (42.34)	333 (34.72)	220 (22.94)	65.86 (13.36)
West Midlands	413 (40.73)	484 (45.23)	586 (54.77)	461 (44.93)	333 (32.46)	232 (22.61)	64.45 (15.38)	399 (42.95)	436 (44.58)	542 (55.42)	354 (37.78)	323 (34.47)	260 (27.75)	65.58 (16.05)
East of England	503 (42.20)	556 (44.80)	685 (55.20)	556 (46.37)	384 (32.03)	259 (21.60)	65.28 (13.34)	514 (46.77)	504 (44.02)	641 (55.98)	438 (39.75)	393 (35.66)	271 (24.59)	65.74 (14.78)
London	333 (40.71)	349 (40.77)	507 (59.23)	404 (49.75)	238 (29.31)	170 (20.94)	63.54 (15.57)	302 (41.26)	322 (41.82)	448 (58.18)	314 (42.43)	255 (34.46)	171 (23.11)	65.08 (15.46)
South East	615 (39.55)	697 (42.97)	925 (57.03)	739 (46.89)	490 (31.09)	347 (22.02)	65.46 (13.06)	639 (44.53)	648 (43.23)	851 (56.77)	569 (38.89)	523 (35.75)	371 (25.36)	66.45 (13.40)
South West	469 (42.95)	504 (44.02)	641 (55.98)	489 (44.01)	354 (31.86)	268 (24.12)	66.14 (13.12)	489 (48.71)	476 (44.99)	582 (55.01)	378 (37.06)	361 (35.39)	281 (27.55)	66.28 (15.16)
	**WAVE 7 (2014–2015)**							**WAVE 8 (2016–2017)**						
North East	212 (50.12)	195 (43.43)	254 (56.57)	132 (30.28)	158 (51.92)	146 (33.49)	68.10 (15.45)	202 (53.72)	172 (43.22)	226 (56.78)	82 (20.97)	161 (41.18)	148 (37.85)	70.39 (13.04)
North West	378 (43.15)	402 (43.41)	524 (56.59)	308 (34.07)	365 (61.21)	231 (25.55)	67.15 (13.72)	370 (48.49)	349 (43.68)	450 (56.32)	205 (26.42)	357 (46.01)	214 (27.58)	68.02 (14.45)
Yorkshire and The Humber	360 (46.51)	339 (42.06)	467 (57.94)	262 (33.04)	318 (59.85)	213 (26.86)	68.19 (11.71)	339 (48.36)	302 (41.20)	431 (58.80)	188 (26.18)	310 (43.18)	220 (30.64)	69.04 (12.69)
East Midlands	351 (42.39)	374 (43.19)	492 (56.81)	296 (35.03)	327 (59.52)	222 (26.27)	67.51 (13.18)	351 (47.56)	331 (43.04)	438 (56.96)	204 (27.20)	330 (44.00)	216 (28.80)	68.64 (13.53)
West Midlands	359 (44.65)	390 (45.56)	466 (54.44)	274 (33.21)	299 (54.23)	252 (30.55)	66.78 (16.46)	340 (48.02)	346 (45.89)	408 (54.11)	203 (28.04)	294 (40.61)	227 (31.35)	67.52 (16.94)
East of England	417 (42.55)	448 (43.62)	579 (56.38)	324 (32.69)	383 (57.39)	284 (28.66)	67.24 (14.55)	423 (47.37)	402 (43.37)	525 (56.63)	218 (24.17)	385 (42.68)	299 (33.15)	68.81 (14.20)
London	258 (40.12)	284 (42.07)	391 (57.93)	239 (36.66)	241 (58.30)	172 (26.38)	66.50 (15.04)	249 (46.03)	239 (42.00)	330 (58.00)	158 (28.42)	223 (40.11)	175 (31.47)	68.83 (13.78)
South East	537 (42.96)	551 (41.90)	764 (58.10)	388 (30.29)	512 (57.32)	381 (29.74)	67.85 (13.94)	508 (46.35)	479 (41.87)	665 (58.13)	253 (22.71)	499 (44.79)	362 (32.50)	69.17 (14.36)
South West	413 (46.67)	411 (43.82)	527 (56.18)	279 (30.79)	348 (55.48)	279 (30.79)	67.59 (15.39)	399 (52.02)	344 (43.05)	455 (56.95)	175 (22.58)	349 (45.03)	251 (32.39)	68.79 (15.24

Values are expressed as column N (%) unless otherwise is indicated

^a^ GOR: Government Office Regions

^b^ Self-reported hearing loss: the sum of those that rated their hearing as fair or poor on a five-point Likert scale (excellent, very good, good, fair or poor), or responded positively in the question whether they find it difficult to follow a conversation if there is background noise (such as TV, radio or children playing)

^c^ Mean (SD): mean age in years (Standard deviation)

Table 2 shows participants’ socio-economic and lifestyle factors and HL prevalence in England in eight Waves of the ELSA*.* In Waves 2–8, the highest prevalence of HL was reported in the GORs that had the highest prevalence of participants belonging to the most deprived quintiles (fifth) according to the IMD. Compared to other GORs, the North East had the highest HL prevalence consistently in all Waves, along with the highest percentage of participants in the most deprived IMD quintile. The rates reached the highest in Wave 7 (2015–2017), with 50.12% of the participants self-reporting HL (95%CI 45.26–54.98) and 39.12% for those residing in an area in the most deprived IMD quintile (95%CI 36.62–41.67).

**Table 2:** Participants’ socioeconomic and lifestyle factors and hearing loss prevalence in England in 8 Waves of English Longitudinal Study of Ageing (ELSA)

GOR^**a**^	Hearing loss^**b**^	Lowest education^**c**^	Manual Occupation^**d**^	Lowest Income^**e**^	Lowest wealth^**f**^	Most deprived^**g**^	Alcohol misuse^**h**^	Hearing loss	Lowest education	Manual Occupation	Lowest Income	Lowest wealth	Most deprived	Alcohol misuse
	**WAVE 1 (2002–2003)**							**WAVE 2 (2004–2005)**						
North East	325 (42.54)	362 (46.89)	404 (53.02)	169 (23.06)	169 (23.87)	261 (33.76)	74 (9.92)	254 (43.34)	249 (42.35)	320 (54.89)	125 (22.52)	148 (26.31)	191 (32.21)	96 (29.91)
North West	603 (38.78)	699 (44.38)	744 (48.00)	296 (20.05)	296 (20.60)	441 (27.96)	179 (11.94)	432 (36.86)	486 (40.77)	550 (46.61)	233 (20.77)	231 (19.52)	298 (24.98)	233 (34.88)
Yorkshire and The Humber	549 (43.06)	596 (46.09)	662 (52.21)	280 (23.12)	280 (23.31)	323 (24.96)	131 (10.62)	401 (40.46)	431 (43.01)	501 (50.86)	226 (24.04)	183 (22.13)	236 (23.51)	175 (30.92)
East Midlands	449 (39.18)	493 (42.25)	552 (47.83)	205 (19.21)	205 (19.18)	158 (13.53)	122 (10.99)	335 (35.52)	396 (41.42)	447 (47.25)	161 (18.34)	175 (19.70)	115 (12.02)	182 (33.46)
West Midlands	452 (36.78)	633 (50.28)	604 (48.99)	261 (22.19)	261 (22.48)	201 (15.97)	130 (10.75)	375 (38.82)	456 (47.06)	458 (48.06)	181 (19.93)	189 (18.83)	145 (14.87)	168 (31.88)
East of England	507 (37.50)	540 (39.82)	539 (40.50)	204 (16.16)	204 (16.20)	57 (4.20)	101 (7.77)	400 (37.00)	398 (36.58)	427 (39.61)	143 (14.19)	137 (17.36)	48 (4.38)	224 (34.46)
London	385 (34.16)	488 (42.69)	445 (40.83)	255 (24.36)	255 (23.94)	237 (20.72)	109 (10.01)	292 (35.39)	328 (39.38)	326 (40.70)	162 (21.15)	189 (16.97)	163 (19.54)	133 (33.84)
South East	681 (36.13)	651 (34.05)	703 (37.45)	261 (14.61)	261 (14.76)	66 (3.45)	215 (11.67)	513 (35.19)	449 (30.61)	513 (35.33)	224 (16.40)	198 (19.18)	46 (3.12)	370 (40.31)
South West	510 (38.37)	483 (35.99)	556 (42.54)	227 (18.17)	227 (17.57)	64 (4.77)	100 (7.82)	420 (39.77)	352 (33.08)	426 (40.69)	186 (18.49)	154 (16.20)	40 (3.75)	213 (34.80)
	**WAVE 3 (2006–2007)**							**WAVE 4 (2008–2009)**						
North East	284 (47.81)	218 (36.33)	323 (54.56)	117 (21.35)	146 (26.64)	204 (33.72)	84 (26.33)	296 (47.21)	225 (34.72)	334 (53.27)	114 (19.29)	143 (24.20)	196 (30.02)	117 (35.03)
North West	458 (40.39)	341 (29.45)	505 (43.95)	209 (20.29)	236 (22.91)	299 (25.75)	198 (31.94)	511 (40.36)	379 (29.04)	520 (41.63)	217 (18.63)	243 (20.86)	288 (21.85)	262 (39.70)
Yorkshire and The Humber	452 (42.68)	372 (34.35)	518 (48.82)	225 (23.20)	186 (19.18)	238 (21.90)	183 (30.86)	491 (44.60)	351 (30.76)	519 (47.18)	224 (21.66)	207 (20.02)	223 (19.44)	207 (34.56)
East Midlands	368 (37.98)	321 (32.59)	434 (44.60)	193 (22.29)	175 (20.21)	116 (11.72)	163 (31.05)	434 (40.49)	357 (32.19)	475 (44.35)	218 (22.13)	172 (17.46)	109 (9.74)	199 (32.52)
West Midlands	392 (39.68)	363 (36.23)	451 (46.02)	168 (18.79)	182 (20.36)	141 (14.03)	170 (31.89)	461 (41.31)	405 (35.31)	502 (45.43)	204 (19.84)	199 (19.36)	188 (16.33)	219 (36.44)
East of England	461 (40.33)	329 (28.41)	447 (39.01)	173 (17.06)	144 (14.20)	49 (4.20)	200 (30.86)	530 (41.15)	374 (28.55)	464 (36.65)	197 (17.00)	166 (14.32)	62 (4.69)	241 (32.66)
London	348 (40.65)	267 (30.76)	319 (37.71)	157 (20.55)	175 (22.91)	152 (17.41)	152 (36.54)	348 (38.71)	266 (28.94)	315 (36.04)	171 (20.90)	201 24.57)	184 (19.78)	159 (35.49)
South East	534 (35.77)	348 (22.77)	512 (33.84)	209 (15.61)	202 (15.09)	50 (3.26)	364 (40.00)	669 (38.45)	399 (22.37)	595 (34.33)	227 (14.39)	199 (12.62)	81 (4.51)	396 (37.71)
South West	453 (43.06)	267 (25.24)	401(38.26)	163 (17.23)	129 (13.64)	33 (3.09)	217 (36.35)	501 (43.00)	283 (23.86)	406 (35.09)	180 (16.73)	148 (13.75)	44 (3.66)	257 (37.63)
	**WAVE 5 (2010–2011)**							**WAVE 6 (2012–2013)**						
North East	295 (51.85)	196 (33.73)	287 (49.91)	99 (18.50)	146 (27.29)	155 (26.27)	116 (38.16)	249 (50.00)	172 (33.46)	239 (46.59)	90 (19.11)	95 (20.17)	142 (27.26)	104 (37.96)
North West	481 (42.68)	317 (27.26)	455 (39.16)	187 (17.76)	229 (21.75)	236 (19.92)	249 (36.89)	471 (46.96)	288 (27.32)	397 (37.59)	171 (18.08)	162 (17.12)	215 (20.11)	212 (38.69)
Yorkshire and The Humber	457 (45.11)	330 (31.88)	491 (48.18)	195 (20.81)	193 (20.60)	197 (18.87)	195 (34.64)	453 (48.81)	288 (30.16)	440 (46.17)	204 (23.53)	149 (17.19)	175 (18.08)	165 (34.74)
East Midlands	399 (39.66)	331 (31.95)	457 (44.59)	226 (24.25)	172 (18.45)	99 (9.37)	163 (30.47)	391 (41.68)	296 (30.58)	410 (42.05)	199 (22.85)	128 (14.70)	101 (10.24)	163 (33.33
West Midlands	413 (40.73)	357 (33.81)	450 (42.98)	214 (22.62)	186 (19.66)	162 (15.14)	216 (38.43)	399 (42.95)	322 (33.23)	404 (41.87)	188 (21.58)	149 (17.11)	150 (15.34)	166 (33.07)
East of England	503 (42.20)	357 (29.24)	456 (37.72)	186 (16.80)	147 (13.28)	61 (4.92)	236 (33.86)	514 (46.77)	303 (26.81)	401 (35.55)	169 (16.41)	125 (12.14)	57 (4.98)	212 (33.76)
London	333 (40.71)	230 (27.19)	290 (35.32)	153 (20.35)	199 (26.46)	156 (18.22)	162 (40.30)	302 (41.26)	200 (26.42)	253 (33.91)	145 (21.17)	148 (21.61)	136 (17.66)	126 (34.81)
South East	615 (39.55)	336 (21.07)	543 (34.24)	220 (15.51)	185 (13.05)	76 (4.69)	367 (38.23)	639 (44.53)	297 (20.14)	473 (31.89)	188 (14.37)	127 (9.71)	70 (4.67)	337 (37.40)
South West	469 (42.95)	249 (22.11)	380 (34.08)	188 (18.29)	162 (15.76)	40 (3.49)	220 (33.79)	489 (48.71)	225 (21.66)	377 (36.04)	166 (17.44)	113 (11.87)	40 (3.78)	200 (34.90)
	**WAVE 7 (2014–2015)**							**WAVE 8 (2016–2017)**						
North East	212 (50.12)	141 (31.76)	204 (46.05)	77 (19.40)	75 (18.89)	575 (39.12)	78 (32.64)	202 (53.72)	114 (29.16)	196 (50.26)	91 (24.73)	42 (11.41)	97 (24.37)	69 (33.82)
North West	378 (43.15)	233 (25.46)	324 (35.41)	157 (19.12)	114 (13.89)	860 (32.72)	190 (39.09)	370 (48.49)	190 (24.11)	289 (36.72)	138 (19.49)	141 (19.92)	151 (18.90)	172 (41.15)
Yorkshire and The Humber	360 (46.51)	234 (29.32)	361 (45.35)	156 (21.64)	120 (16.64)	715 (30.59)	126 (30.88)	339 (48.36)	209 (29.11)	354 (49.65)	151 (23.20)	95 (14.59)	140 (19.10)	108 (30.34)
East Midlands	351 (42.39)	242 (28.40)	350 (40.89)	168 (21.73)	119 (15.39)	440 (19.29)	154 (34.00)	351 (47.56)	205 (27.22)	306 (40.53)	155 (22.33)	126 (18.16)	78 (10.14)	118 (31.13)
West Midlands	359 (44.65)	262 (30.82)	350 (41.22)	173 (22.97)	102 (13.55)	670 (27.66)	142 (33.81)	340 (48.02)	209 (28.21)	309 (41.59)	147 (22.24)	106 (16.04)	112 (14.85)	130 (33.94)
East of England	417 (42.55)	257 (25.42)	343 (33.93)	134 (14.66)	84 (9.19)	250 (10.92)	180 (33.27)	423 (47.37)	216 (23.89)	321 (35.51)	127 (15.19)	201 (24.04)	52 (5.61)	174 (34.05)
London	258 (40.12)	170 (25.41)	215 (32.98)	112 (19.15)	118 (20.17)	575 (27.43)	103 (34.56)	249 (46.03)	134 (24.06)	183 (33.33)	98 (19.41)	113 (22.38)	79 (13.88)	86 (33.20)
South East	537 (42.96)	242 (18.63)	411 (31.62)	177 (15.43)	112 (9.76)	275 (10.00)	259 (34.35)	508 (46.35)	198 (17.66)	356 (31.62)	181 (17.73)	299 (29.29)	46 (4.02)	221 (34.05)
South West	413 (46.67)	178 (19.22)	326 (35.09)	138 (16.51)	84 (10.05)	160 (6.88)	165 (33.60)	399 (52.02)	141 (18.08)	254 (32.52)	139 (19.39)	201 (28.03)	43 (5.38)	146 (34.11)

^a^ GOR: Government Office Regions

^b^ Self-reported hearing loss: the sum of those that rated their hearing as fair or poor on a five-point Likert scale (excellent, very good, good, fair or poor), or responded positively in the question whether they find it difficult to follow a conversation if there is background noise (such as TV, radio or children playing)

^C^ No qualifications

d Manual occupations

e Income categories in quantiles

f Wealth categories in quantiles

g IMD: Index of Multiple Deprivation (in quintiles)

h Alcohol intake above the low-risk level guidelines (> 14 units/week)

Moreover, the highest prevalence of HL was reported in the GORs with the highest prevalence of participants belonging in the group of routine or manual occupations. In Waves 1–5, participants from the North East had both the highest rates of routine or manual occupations and the highest prevalence rates of HL among all GORs.

Finally, we observed an increasing trend over time in total alcohol misuse (alcohol consumption above the low-risk level guidelines) in all Waves; the prevalence of alcohol misuse increased in 2002–2017, going from an average of 10.17% in Wave 1 to 33.98% in Wave 8. The South West had one of the highest prevalence rates of alcohol misuse, in parallel with one of the highest prevalence rates of self-reported HL. It is worth mentioning that their sample was of a higher SEP in all Waves (with respect to education, occupation, income, wealth and IMD).

**Fig. 1:**
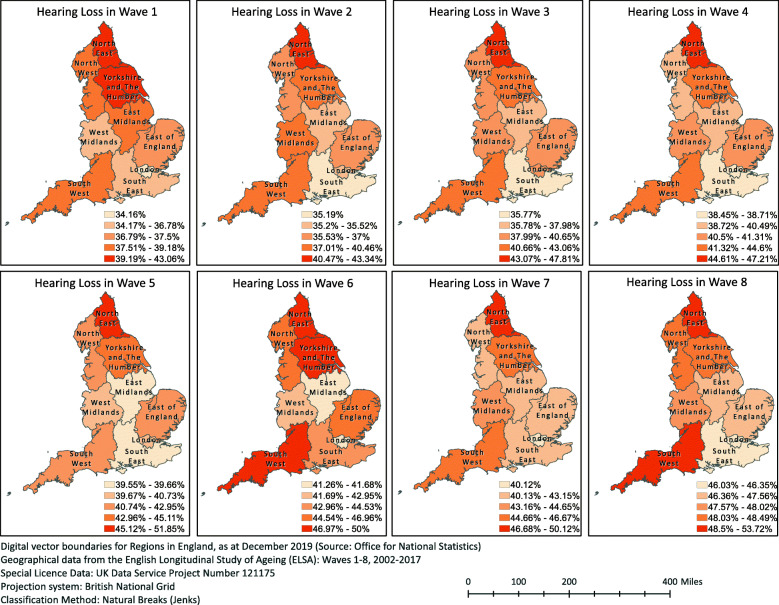
Map of England by Government Office Regions, showing prevalence rates of self-reported hearing loss in eight Waves of the English Longitudinal Study of Ageing (ELSA). This work by Dialechti Tsimpida is licensed under a Creative Commons Attribution 4.0 International License.

Figure 1 illustrates the prevalence of HL in each GOR across the eight Waves of the ELSA. There was an increasing trend over time in the HL prevalence for all five classes. In samples of significantly equal means of age between GORs, the mean HL prevalence increased from 38.50 (95%CI 37.37–39.14) in Wave 1 to 48.66 (95%CI 47.11–49.54) in Wave 8.

**Fig. 2:**
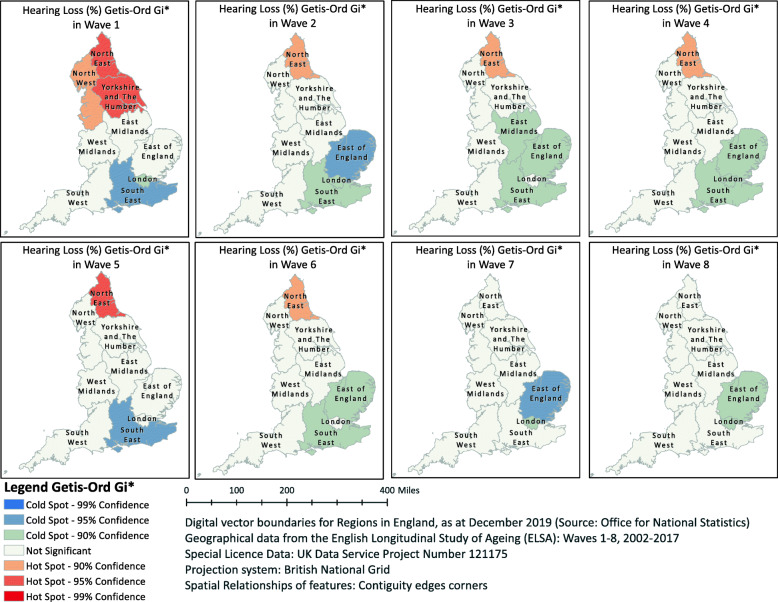
Map of England by Government Office Regions showing the spatial clusters of hearing loss prevalence according to Hot Spot and Cold Spot analyses ^a^ using the Getis-Ord Gi* statistic in eight Waves of the English Longitudinal Study of Ageing (ELSA). ^a^ The Hot Spots and Cold Spots indicate unexpected spatial spikes of high or low values, respectively, showing that the distribution of these values in the dataset is more spatially clustered than would be expected if underlying spatial processes were truly random. This work by Dialechti Tsimpida is licensed under a Creative Commons Attribution 4.0 International License.

Figure 2 depicts the Hot Spot and Cold Spot analyses in England, based on the Getis-Ord Gi* statistic; the analyses identified statistically significant spatial clusters of high values (Hot Spots) and low values (Cold Spots) in all Waves of the ELSA. We observed some statistically significant spatial clusters of HL prevalence covering specific GORs in England as all Hot and Cold Spots were found in the northern and southern parts of England, respectively. In essence, we observed spatial clustering of high (Hot) or low (Cold) values that were more pronounced than one would expect in a random distribution of these same values. In Waves 1–6, the z-score value in the North East GOR was positive, which means that the spatial distribution of high values in this part of England was more spatially clustered than would be expected if the underlying spatial processes were truly random. On the other hand, during the same period the z-score value in the South East GOR was negative, which means that the spatial distribution of low values in the dataset was more spatially clustered than would be expected if the underlying spatial processes were truly random.

**Fig. 3:**
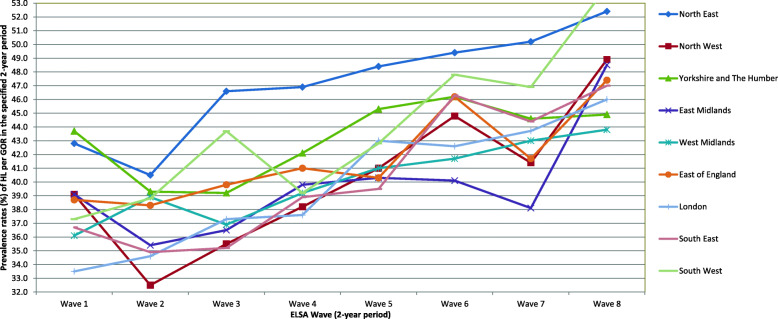
Predicted probabilities and 95% Confidence Intervals of hearing loss (HL) prevalence at Regions of England in eight Waves of the English Longitudinal Study of Ageing (ELSA) ^a, b^. ^a^ The x-axis refers to ELSA Wave (Wave 1: 2002–3, Wave 2: 2004–5, Wave 3: 2006–7, Wave 4: 2008–9, Wave 5: 2010–11, Wave 6: 2012–13, Wave 7: 2014–15, Wave 8: 2016–17), and the y-axis refers to prevalence rates of HL per GOR in the specified 2-year period. ^b^The factor variables (age, sex, education, occupation, income, wealth, IMD and alcohol consumption) were hold at their means for each ELSA Wave.

Figure 3**.** shows the predicted probabilities of HL prevalence in each region and Wave of the ELSA, holding all other variables in the model at their means. The results tell us that if we had two otherwise-average individuals in each Wave, the probability of them having HL would vary significantly among regions. For example, in Wave 1, one’s probability of having HL in Yorkshire and the Humber would be 10.2% higher than it would be for an otherwise-comparable participant in London (Yorkshire and the Humber APM = .437, London APM = .335, MEM = .437–.335 = .102) (please also see Additional File 1). The predicted probability of having HL demonstrated an increasing trend over time in all regions. The maximum increase of predicted HL probability among older adults of significantly equal age in the 15-year period was in the South West, which had a 45% increase (Wave 1:37.3 [34.4–40.2], Wave 8: 54.1 [48.9–59.2]).

## Discussion

### Summary of main findings

In this study, we examined the regional patterns and trends of HL prevalence in England in the ELSA over 15 years (2002–2017). We found that among samples with equal means of age, there was a 15-year increasing trend in HL prevalence in all five classes. The mean HL prevalence increased from 38.50 (95%CI 37.37–39.14) in Wave 1 to 48.66 (95%CI 47.11–49.54) in Wave 8. We identified three critical patterns of findings concerning regional trends: the highest HL prevalence among samples with equal means of age was observed in GORs with the highest prevalence of participants (a) in the most deprived (IMD) quintile (fifth), (b) in routine or manual occupations and (c) that misused alcohol, irrespective of SEP. The APMs for HL showed marked regional variability and evidence of a North–South divide.

### Comparison with previous literature

Previous research has utilised geographical indices representing social and material disadvantages for identifying health inequalities [7]. Our study provided evidence for the existence of sociospatial inequalities in HL, adding to our previous work that challenged the existing conceptualisation of HL as an inevitable accompaniment of growing old [8]. Globally, there is a dramatic increase in HL cases, going from 42 million people in 1985 to about 360 million in 2011 and over 466 million in 2019 [28]. Our study presented a similar increase pattern but also showed that the increase in HL prevalence is not related to the ageing of the population, as widely believed [29, 30], but could potentially be due to social and lifestyle changes in the population [31]. Supporting our assumption, a previous study found a decline in HL prevalence among US adults aged 20–69 from the 2011–2012 cycle of the US National Health and Nutrition Examination Survey when compared to participants from the previous decade [32]. The explanation given by the authors for the declining prevalence was a reduction in exposure to occupational noise and the beneficial lifestyle changes of the participants, though that population study is not comparable to the ELSA cohort.

In our study, a North-South divide was revealed in hearing health inequalities that was previously unknown. The North-South gap is not surprising, as there is a significant history of socio-economic and health disparities between Northern and Southern England [33, 34]. The higher rates of unemployment and no qualifications in the North than in the South are in line with previous research in England [35]. We also found that alcohol misuse was high in areas with a high prevalence of HL, such as the South West, which over time developed one of the highest prevalence rates of alcohol misuse despite its higher socio-economic status compared to other GORs. This finding supports a previous study on the ELSA that found that alcohol intake above the low-risk-level guidelines [23] was significantly associated with HL among older adults in England, along with socio-economic factors [8]. However, the findings from this study indicate that the relationship between SEP and drinking habits is rather complicated; the last statistical release on adult drinking habits in Great Britain showed that those in managerial and professional occupations drink alcohol in higher proportions compared to those in routine and manual occupations. In addition, similarly to our study, it was found that the South East GOR, when compared to other GORs in England, had a higher proportion of adults drinking alcohol the week before the interview [36].

### Strengths and limitations

This is the first study to investigate the geographical patterns and trends of HL in a representative cohort of older adults and among adults in general. The findings provide evidence that HL has increased over time, but the increasing trend in HL prevalence is not *age-related*, as widely believed. We found wide variation in HL prevalence in representative samples from different regions in England that had similar age profiles, and the increase rate of HL ranged from 3.2 to 45%. Thus, the strengths of this study are that HL is highlighted as an increasingly important public health problem in England and a spatial dimension is added to the evidence for the association of socio-economic and lifestyle determinants of HL among samples of older adults.

However, there are also important limitations. First, the unit of our analyses (in GORs) had a low geographic resolution, which introduces uncertainty in the observed relations and may fail to reveal geographic details that we could notice with smaller geographic units. Moreover, it was not possible to perform geographically weighted regression analyses; a minimum of 30 input features is required (instead of nine GORs) to explore the relationships between the areas’ socio-economic characteristics and HL prevalence. Furthermore, the ELSA’s size is regarded as too small to conduct geographic analysis on a larger scale, as numerous participants would be required in each unit.

Future research should build on this analysis using small area statistics (such as Lower Layer Super Output Areas) and investigate more localised patterns and determinants of place-to-place HL differences in England [35]. Such research would help to quantify potential ‘area effects’ on hearing health outcomes, allowing for generalisable results of spatial associations with HL rates. Moreover, the research could help to separate the role of proxies of areas (such as area deprivation) to individual-level determinants of HL (such as lifestyle behavioural choices), as individual choices are rooted in the broader social and economic structural contexts [31].

We were aware that the self-reported measures of HL in the ELSA might underestimate the real HL outcomes; for this reason, we conducted additional work to examine the validity of self-reported data through comparisons with the findings of objective HL measures available only in Wave 7 of the ELSA. We found that the self-reported measures correctly classified seven in every ten people with objectively assessed HL [16]. However, for the scope of our analyses, we assumed the available hearing measure as a suitable indicator of HL.

Another limitation is that the ELSA concentrates on individuals living in private households, so individuals living in institutions (e.g. residential and nursing homes) are not included in the samples [15]. Furthermore, ELSA does not capture the type of HL; future analyses examining types of HL would add important value.

Finally, the domains of IMD are not provided with the ELSA geography file, thereby not allowing further exploration. There was a small number of respondents moving to a different area between Waves, which resulted in an associated change in the IMD quintile [14]. However, a similar number of respondents experienced an increase or a decline in their IMD quintile, and the total numbers of movers did not exceed 1% for any Wave [14]; thus, we concluded that this would be unlikely to affect the validity of our findings.

### Research and policy implications

According to the Global Burden of Disease Study, HL is the third leading cause of years lived with disability in England [37], and accurate prevalence estimates are needed to inform the strategic planning of hearing health policy and health services. To date, the prevalence of HL estimates in the UK is still based on the Medical Research Council National Hearing Study [3]. In addition, the NHS England has recently published the NHS Hearing Loss data tool [38], which provides estimates of the number of people with HL between 2015 and 2035 in order to help organisations plan services on local authority (LA) and CCG levels. However, according to our study, the above tool is inappropriate for estimating the number of people with HL; this study showed that in a representative cohort, there were important differences across different regions in England, which contradicts the Hearing in Adults study that did not find differences across the only four British cities that it was based on (Cardiff, Glasgow, Nottingham and Southampton) [3].

HL has affected a markedly larger proportion of the UK population in 2002–2017. The high levels of spatial clustering for hearing-related outcomes have significant implications for the planning of health services, including the availability of access to hearing aids. The high-risk regions in England must be expansively recognised based on their spatiotemporal HL profiles [39]. This kind of spatial evidence could provide commissioners with robust data based on actual needs, rather than inaccurate estimates of HL prevalence. Such prior knowledge could potentially have altered the North Staffordshire CCG’s decision in 2015 to end the routine free provision of hearing aids for people with mild or moderate HL in their area of duty [40], where according to our analyses, the burden of HL is greatest. This study revealed, therefore, the potential risks from the paucity of robust epidemiological hearing data, which are needed now as much as ever to increase understanding of the impact of social, financial and personal health advantages on HL across the life course [1].

The findings from the time-series analyses in this manuscript might encourage HL preventive strategies, including interventions to promote ‘healthier lifestyles’ and targeted interventions in areas where there are high levels of deprivation clustering. Future research should also explore spatiotemporal diffusion patterns in the ELSA’s international sister studies to acquire a global perspective of socio-spatial inequalities in hearing health.

## Conclusions

We have identified elevated social and geographical patterning of trends in HL; different levels of exposure to socio-economic and lifestyle factors lead to geographical hearing health variation among English populations of significantly equal age. The socio-economic, lifestyle and regional patterns and trends in HL support the argument that the increase of HL is not ‘*age-related*’, as widely believed, and HL, therefore, might be a highly preventable lifestyle-related condition.

These findings also point to the need for a stronger health policy response. According to the inextricable link of health and geography, the regional variation in hearing health outcomes should be examined for health policy decisions according to spatial needs. The audiological services may need to be redesigned to take socio-economic and lifestyle risk factors for HL into account in order to prevent the further exacerbation of inequalities in regions with spatial hearing health inequality.

## Supplementary Information

**Additional file 1: Table 1** and **Table 2**. One-Way ANOVA results of means of age at Regions of England in eight Waves of the English Longitudinal Study of Ageing (ELSA) and Predicted probabilities and 95% Confidence Intervals of hearing loss prevalence at Regions of England in eight Waves of the English Longitudinal Study of Ageing (ELSA).

## Data Availability

The English Longitudinal Study of Ageing dataset is publicly available via the UK Data Service (http://www.ukdataservice.ac.uk). The geographical variables were provided to the first author under a Special License and Secure Access agreement (UK Data Service Project Number: 121175), and so are not publicly available. Statistical code is available from the corresponding author upon reasonable request at dialechti.tsimpida@manchester.ac.uk.

## Abbreviations

APMs: Predictions at the Means
CCGs: Clinical Commissioning Groups
ELSA: English Longitudinal Study of Ageing
GOR: Geographical Office Regions
HL: Hearing loss
HSE: Health Survey for England
IMD: Index of Multiple Deprivation
NS-SEC: National Statistics socio-economic classification
PAF: Postcode Address File
SEP: Socioeconomic position
