# Supplementary material for: Regional patterns and trends of hearing loss in England: evidence from the English longitudinal study of ageing (ELSA) and implications for health policy
Source: BMC Geriatr. 2020 Dec 15;20:536. doi: 10.1186/s12877-020-01945-6 (PMC7737370; doi:10.1186/s12877-020-01945-6)
Supplement: Supplementary file 1 — Additional file 1: Table 1 and Table 2. One-Way ANOVA results of means of age at Regions of England in eight Waves of the English Longitudinal Study of Ageing (ELSA) and Predicted probabilities and 95% Confidence Intervals of hearing loss prevalence at Regions of England in eight Waves of the English Longitudinal Study of Ageing (ELSA). [file 12877_2020_1945_MOESM1_ESM.docx]

| **Additional File**  **Table 1**. One-Way ANOVA results of means of age at Regions of England in eight Waves of the English Longitudinal Study of Ageing (ELSA) | | | | | | | | |
| --- | --- | --- | --- | --- | --- | --- | --- | --- |
|  | **Source** | **Degrees of Freedom DF** | **Sum of Squares SS** | **Mean Square MS** | **F-Stat** | **P-Value** | **Bartlett’s test for equal variances** | **Prob>chi2** |
| **Wave 1**  **(2002/3)** | Between Groups | \|  \| \| --- \|   8 | 2681.90354 | 335.237942 | 2.74 | 0.0052 | 11.4703 | 0.17 |
|  | Within Groups | 11,818 | 1448107.05 | 122.54439 |  |  |  |  |
| **Wave 2**  **(2004/5)** | Between Groups | 8 | 1733.55958 | 216.694947 | **1.90** | **0.0557** | 14.2375 | 0.07 |
|  | Within Groups | 9,184 | 1048209.21 | 114.146707 |  |  |  |  |
| **Wave 3**  **(2006/7)** | Between Groups | 8 | 3982.75449 | 497.844312 | 3.83 | 0.0002 | 16.0442 | 0.04 |
|  | Within Groups | 9,481 | 1231740.12 | 129.916688 |  |  |  |  |
| **Wave 4**  **(2008/9)** | Between Groups | 8 | 2812.61074 | 351.576342 | 3.17 | 0.0014 | 8.1569 | 0.41 |
|  | Within Groups | 10,630 | 1178966.28 | 110.90934 |  |  |  |  |
| **Wave 5**  **(2010/11)** | Between Groups | 8 | 5725.07332 | 715.634165 | 3.97 | 0.0001 | 124.8517 | <0.001 |
|  | Within Groups | 9,801 | 1766317.22 | 180.218062 |  |  |  |  |
| **Wave 6**  **(2012/13)** | Between Groups | 8 | 2399.91545 | 299.989431 | **1.49** | **0.1547** | 130.4451 | <0.001 |
|  | Within Groups | 8986 | 1807938.53 | 201.217421 |  |  |  |  |
| **Wave 7**  **(2014/15)** | Between Groups | 8 | 1984.25469 | 248.031836 | **1.20** | **0.2933** | 129.6364 | <0.001 |
|  | Within Groups | 7,850 | 1619949.92 | 206.389339 |  |  |  |  |
| **Wave 8**  **(2016/17)** | Between Groups | 8 | 2916.22388 | 364.527985 | **1.76** | **0.0795** | 86.7473 | <0.001 |
|  | Within Groups | 6,884 | 1424110.07 | 206.902524 |  |  |  |  |
| **Across all Waves** | Between Groups | 7 | 138.508 | 19.787 | **0.12** | **0.996** | 621.81893 | <0.001 |
|  | Within Groups | 64 | 10,148.390 | 158.569 |  |  |  |  |

**Table 2.** Predicted probabilities and 95% Confidence Intervals of hearing loss prevalence at Regions of England in eight Waves of the English Longitudinal Study of Ageing (ELSA)*

|  | **Wave 1** | **Wave 2** | **Wave 3** | **Wave 4** | **Wave 5** | **Wave 6** | **Wave 7** | **Wave 8** | **increase Wave 1-8** |
| --- | --- | --- | --- | --- | --- | --- | --- | --- | --- |
| **North East** | 42.8 (38.9-46.7) | 40.5 (34.6-46.4) | 46.8 (40.8-52.8) | 46.9 (41.0-52.8) | 48.5 (42.2-54.7) | 49.6 (43.1-56.1) | 50.1 (43.1-57.1) | 52.5 (45.1-59.9) | 22.7% |
| **North West** | 39.1 (36.4-41.8) | 32.5 (28.7-36.4) | 35.6 (31.4-39.8) | 38.2 (34.0-42.3) | 41.1 (37.0-45.2) | 44.8 (40.2-49.4) | 41.2 (36.5-46.0) | 49.1 (43.8-54.4) | 25.6% |
| **Yorkshire and The Humber** | 43.7(40.7-46.7) | 39.3 (35.0-43.7) | 39.3 (34.9-43.6) | 42.1 (37.8-46.5) | 45.3 (40.8-49.9) | 46.3 (41.3-51.2) | 44.5 (39.2-49.8) | 45.1 (39.4-50.8) | 3.2% |
| **East Midlands** | 39.1 (36.0-42.3) | 35.2 (30.9-39.5) | 36.3 (31.8-40.8) | 39.8 (35.5-44.0) | 40.2 (35.6-44.8) | 40.0 (35.3-44.8) | 38.1 (33.3-43.0) | 48.6 (43.1-54.1) | 24.3% |
| **West Midlands** | 36.1 (33.1-39.1) | 38.7 (34.2-43.2) | 36.8 (32.3-41.3) | 39.0 (34.7-43.4) | 41.0 (36.5-45.5) | 41.6 (36.8-46.3) | 43.0 (37.7-48.2) | 43.8 (38.3-49.3) | 21.3% |
| **East of England** | 38.7 (35.8-41.6) | 38.3 (34.2-42.4) | 39.8 (35.6-44.1) | 41.1 (37.1-45.0) | 40.3 (36.2-44.3) | 46.1 (41.7-50.4) | 41.7 (37.1-46.2) | 47.4 (42.6-52.2) | 22.5% |
| **London** | 33.5 (30.4-36.7) | 34.6 (29.4-39.7) | 37.1 (31.9-42.2) | 37.7 (32.6-42.7) | 43.0 (37.6-48.4) | 42.8 (37.1-48.5) | 43.8 (37.4-50.1) | 45.7 (38.9-52.6) | 36.4% |
| **South East** | 36.7 (34.2-39.1) | 35.1 (31.6-38.5) | 35.3 (31.8-38.8) | 38.9 (35.5-42.3) | 39.5 (36.1-43.0) | 46.5 (42.7-50.2) | 44.4 (40.4-48.5) | 46.9 (42.6-51.3) | 27.8% |
| **South West** | 37.3 (34.4-40.2) | 38.7 (34.5-42.9) | 43.6 (39.2-48.0) | 39.1 (35.0-43.1) | 42.5 (38.3-46.7) | 47.5 (43.0-52.0) | 47.1 (42.3-51.9) | 54.1 (48.9-59.2) | 45.0% |

* Holding factor variables (age, gender, education, occupation, income, wealth, IMD and alcohol consumption) at their means for each ELSA Wave.
